# Chemical and Microscopical Diagnosis

**Published:** 1910-01

**Authors:** 


**Chemical and Microscopical Diagnosis.** By **Francis Carter Wood,** M.D., Professor of Clinical Pathology, College of Physicians and Surgeons, New York. Octavo, pp. 771. Illustrated. New York and London: D. Appleton & Co. 1909. (Cloth, $5.00.)

In the revision of his book for a second time, the author has made an effort to keep the text as simple and clear as possible, that it may be better understood by its readers who have had no extensive laboratory training. In this he has succeeded excellently well, and has imparted a vast amount of information in a very readable manner.

The work shows a careful and painstaking revision. Recent studies relative to the blood, have necessitated changes in the chapter dealing with that subject. Sections have also been added on the new tuberculosis reactions, on the opsonic index and on the Wassermann tests for syphilis, all of which will be of much value as aids in diagnosis.

The book is a valuable one, and written by a recognised authority. J. A. R.

